# Supplementary figures and images for: Bacillus velezensis RC116 Inhibits the Pathogens of Bacterial Wilt and Fusarium Wilt in Tomato with Multiple Biocontrol Traits
Source: Int J Mol Sci. 2023 May 10;24(10):8527. doi: 10.3390/ijms24108527 (PMC10217916; doi:10.3390/ijms24108527)

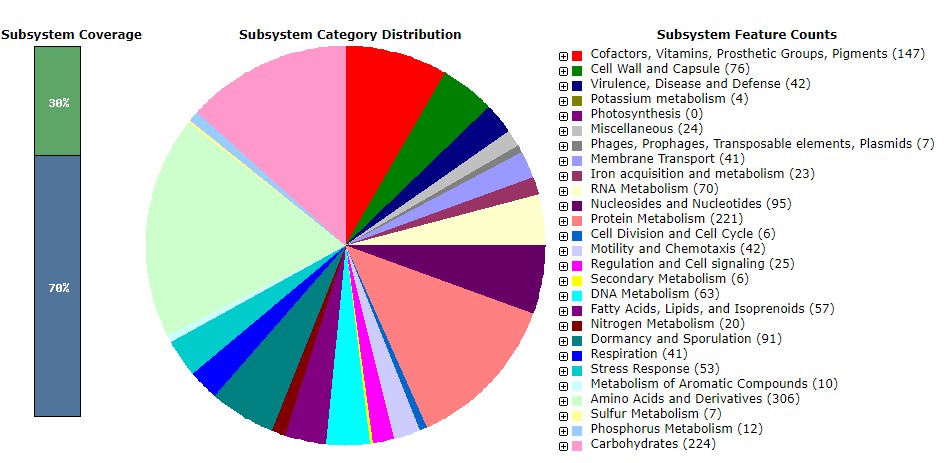

Supplement: Supplementary file 1 [file ijms-24-08527-s001.zip › Supplementary Figure S2.jpg]

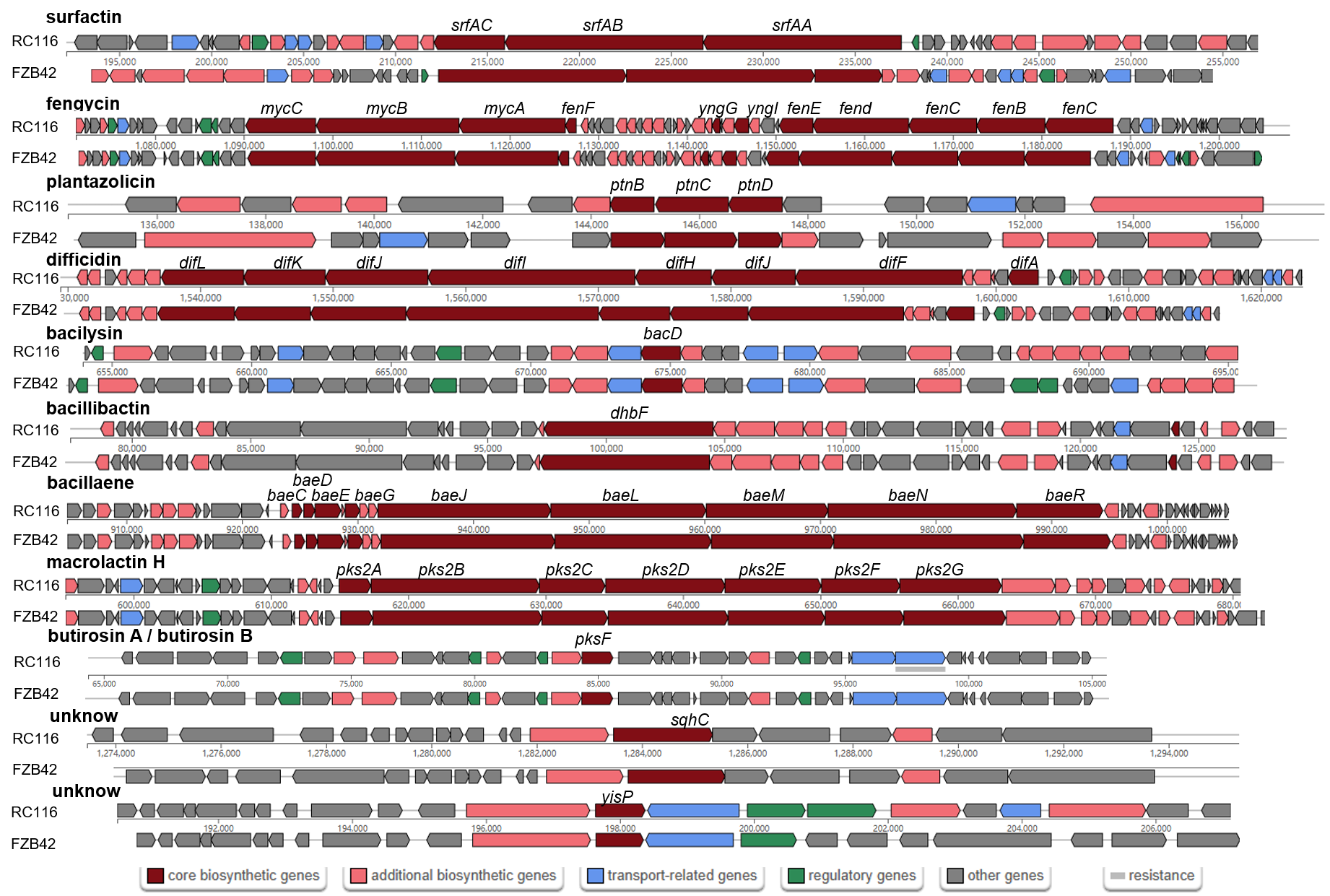

Supplement: Supplementary file 1 [file ijms-24-08527-s001.zip › Supplementary Figure S3.tif]

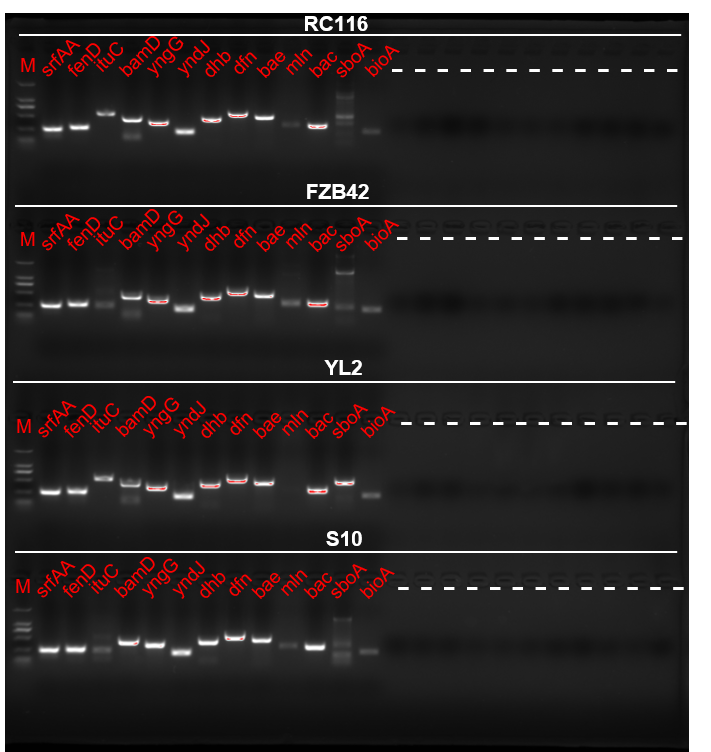

Supplement: Supplementary file 1 [file ijms-24-08527-s001.zip › Supplementary Figure S4.tif]
